# Secondary spontaneous pneumothorax as post-COVID-19 sequela

**DOI:** 10.11604/pamj.2021.39.190.30473

**Published:** 2021-07-08

**Authors:** Moli Jai Jain, Vaishnavi Dilip Yadav

**Affiliations:** 1 Department of Cardiovascular and Respiratory Physiotherapy Sciences, Ravi Nair Physiotherapy College, Datta Meghe Institute of Medical Sciences, Sawangi, Wardha, Maharashtra, India,

**Keywords:** Secondary spontaneous pneumothorax, COVID-19, post-COVID-19 sequela

## Image in medicine

A 48-year-old male patient with a significant history of hypertension for 8 years and newly diagnosed diabetes was admitted on account of fever and difficulty in breathing from 15 days, initially exertional but later present at rest. A nasopharyngeal swab taken for reverse transcription polymerase chain reaction (RT-PCR) testing was positive for COVID-19. Following admission, he was placed on 15 L O_2_/min because of not maintaining saturation later put on a mechanical ventilator on pressure support mode with positive end-expiratory pressure (PEEP) 8 cm H_2_O and 80% FiO_2_ for the next 8 days. High-resolution computed tomography (HRCT) thorax (A) revealed multiple areas of ill-defined ground-glass opacities with septal thickening and few areas of consolidation. There are multiple air-filled cystic spaces (red arrow) in sub-pleural spaces of lateral segment of right middle and lower lobes there is evidence of fibro-bronchiectatic changes as a symptom of post-COVID sequela with computed tomography (CT) severity score 23/25 (severe) and COVID-19 reporting and data system 6 (CO-RADS-6). Later he was put on bilevel positive airway pressure (BiPAP) support for 19 days and gradually weaned off to 12L O_2_/ min via face mask connecting to re-breathing bag. Soon he developed secondary spontaneous Pneumothorax visible on chest X-ray (B) because of which pig tail inter-costal drainage was done in 4^th^ intercostal space in anterior axillary line. Post-COVID-19 complications are become more evident and chest imaging plays an important role in early screening and monitoring such cases.

**Figure 1:**
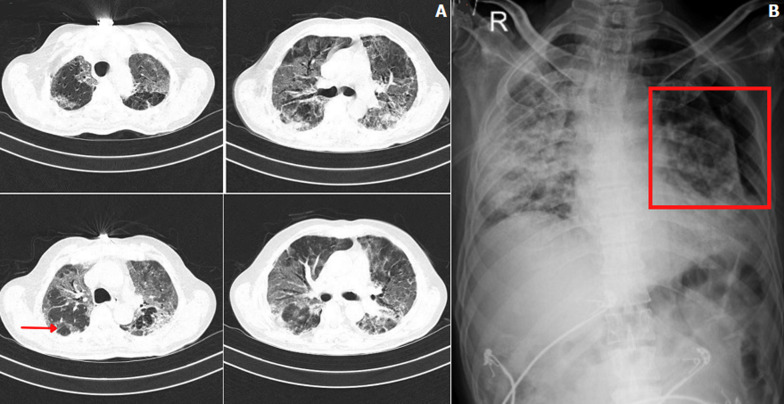
(A) HRCT thorax showing multiple air-filled cystic spaces (red arrow) in sub-pleural spaces of lateral segment of right middle and lower lobes with CT severity score 23/25; (B) chest X-ray showing secondary spontaneous pneumothorax

